# Bioanalytical method development, *in-vivo* pharmacokinetic evaluation, *ex-vivo* platelet aggregation inhibition activity of a novel solid dispersion formulation of ticagrelor

**DOI:** 10.3389/fmedt.2025.1499189

**Published:** 2025-02-06

**Authors:** Abhishek Srivastava, Simrata Bedi, Abhishesh Kumar Mehata, Datta Maroti Pawde, Ketan Vinayakrao Hatware, Mohammad Ahmad Khan, M. S. Muthu, Uma Bhandari

**Affiliations:** ^1^ Formulation Research and Development, Sun Pharmaceutical Industries Ltd, Gurugram, India; ^2^ Department of Pharmacology, SPER, Jamia Hamdard University, New Delhi, India; ^3^ Department of Pharmaceutical Engineering and Technology, IIT, BHU, Varanasi, India; ^4^ Department of Pharmaceutical Sciences, School of Health Sciences and Technology, Dr. Vishwanath Karad MIT World Peace University, Pune, India; ^5^ School of Pharmacy & Technology Management, SVKM’S NMIMS Deemed-to-be University, Shirpur, India

**Keywords:** ticagrelor, antithrombotic agent, solid dispersion, bioavailability, pharmacokinetic

## Abstract

**Background:**

Ticagrelor, a potential antithrombotic drug indicated for cardiovascular events with acute coronary syndrome, has been restricted from its oral use due to poor aqueous solubility. The present investigation aimed to develop validated bioanalytical method for the analysis of plasma samples for improving the oral bioavailability of Ticagrelor. Additionally, evaluation of the improved antiplatelet activity of the Ticagrelor formulation compared to the marketed formulation.

**Methods:**

A bioanalytical method was developed in rat plasma samples using the isocratic separation mode. Plasma samples were processed by liquid-liquid extraction and analyzed by using reverse phase HPLC. A validated method was used for evaluating the pharmacokinetic profile of the developed formulation and marketed formulation in Sprague Dawley rats. Additionally, the ex-vivo antiplatelet aggregation activity was evaluated.

**Results:**

The developed method was accurate and linear (100 ng−800 ng) to quantify the drug in plasma. *An in-vivo* pharmacokinetic study was conducted for formulation at 10 mg/kg and different pharmacokinetic parameters were evaluated. From the results, we observed∼64% enhancements in the oral bioavailability of the Ticagrelor relative to the marketed formulation. The developed formulation (SD1) showed more significant inhibition of ADP-induced platelet aggregation compared to the marketed ticagrelor (RLD) formulation.

**Conclusion:**

In conclusion, we have successfully developed a validated analytical method for estimating Ticagrelor plasma concentration. Additionally, our study successfully enhanced Ticagrelor's oral bioavailability, and the developed formulation has more significant inhibition of ADP-induced platelet aggregation relative to the marketed formulation, indicating its substantial therapeutic potential.

## Graphical Abstract

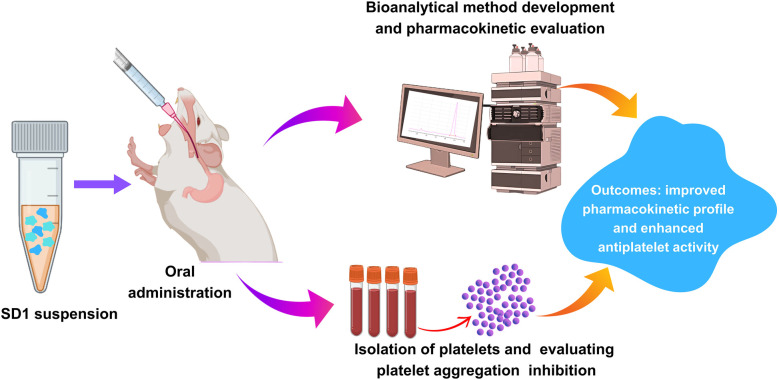

## 1 Introduction

Arterial thrombosis is a critical factor for contributing various cardiovascular events such as ischaemic stroke, myocardial infarction etc. Nowadays, cardiovascular disease is a leading cause of death and morbidity in patients in developed countries (1). Ticagrelor (TC) is a P2Y12 receptor inhibitor used in patients with a history of myocardial infarction or acute coronary syndrome (ACS) to prevent future myocardial infarction, stroke, and particularly to those who are at the risk of cardiovascular death (2). TC belongs to BCS class IV drug with low solubility and permeability (3). TC marketed formulation “Brilinta”, approved by the FDA in 2011, and it popularly became a choice drug in atherothrombotic cardiovascular events (4). The Solid dispersion formulation is envisioned to show a better dissolution profile, resulting in increased bioavailability (5). The proposed formulation with better patient compliance is developed to bring into the market (6). Several literature reports suggested, at present ≈40% of the new chemical entities (NCEs) generated during drug development are lipophilic in nature and undergo problems like erratic absorption, low solubility, poor oral bioavailability associated with pharmacokinetic variations, lack of dose proportionality etc (7).

Despite the widespread use of TC for clinical use, an accurate, validated, and sensitive bioanalytical method is required for estimating the drug concentration in biological matrices such as plasma. This is critical for understanding the pharmacokinetic profile and adjusting the dosing of the drug, particularly in patients with compromised renal function or higher bleeding risk. Presently, available HPLC methods for TC analysis were predominantly focused on the pharmacokinetic analysis or quality control testing of the formulations, which often involved complexity in sample preparations, low sensitivity, and limited applicability in the preclinical and clinical applications (8, 9). In our previous study, Srivastava et al, observed that the drug release profile of the reported optimized formulation was found to be 70.0%, 55.4%, 35.5%, and 30.0% at 90 min, while the reference product showed a release of 9.4%, 20.7%, 8.4%, and 7.8% at 90 min in water, 0.1 N HCl, pH 4.5 acetate buffer and pH 6.8 phosphate buffer, respectively. Therefore, it is evident that the drug is released through diffusion from the polymer matrix (10). However, pharmacokinetic and antiplatelet activity was not evaluated in the animal model.

In this research, we have developed a validated bioanalytical method for TC and performed the *in-vivo* pharmacokinetic study of our developed formulation and marketed TC tablet. This study is essential mainly because it addressed a significant gap in the current bioanalytical approaches by developing validated sensitive, reproducible, robust method for quantifying drug samples in plasma. The novelty of this research includes enhanced sensitivity, selectivity, simple sample preparation method, short run time, and superior resolution.

Further, inhibition of platelet aggregation activity was evaluated in the rat model, which provided comprehensive insights into the therapeutic potential of TC.

## 2 Methods

### 2.1 Preparation of the calibration curve of the TC by HPLC method

5 mg of the TC was dissolved in the 5 ml of the methanol to form 1,000 µg/ml solution.

(stock I); Further, 0.5 ml of the stock I was diluted up to 5 ml with methanol to form the concentration of 100 µg/ml (stock II). Moreover, 0.5 ml of stock II was diluted up to 5 ml with methanol to form 10 µg/ml (stock III). Similarly, 0.5 ml of stock III was diluted up to 5 ml with methanol to form 1 µg/ml or 1,000 ng/ml (Stock IV) (11). The final spiking of the drug from stock IV in isolated plasma, as presented in Table 1.

**Table 1:** Represents sample preparation for the calibration curve.

TC concentration (ng/ml)	Dilution from stock IV	Solvent
100	50–500 µl	Plasma
200	100–500 µl	Plasma
400	200–500 µl	Plasma
600	300–500 µl	Plasma
800	400–500 µl	Plasma

**Figure 1:**
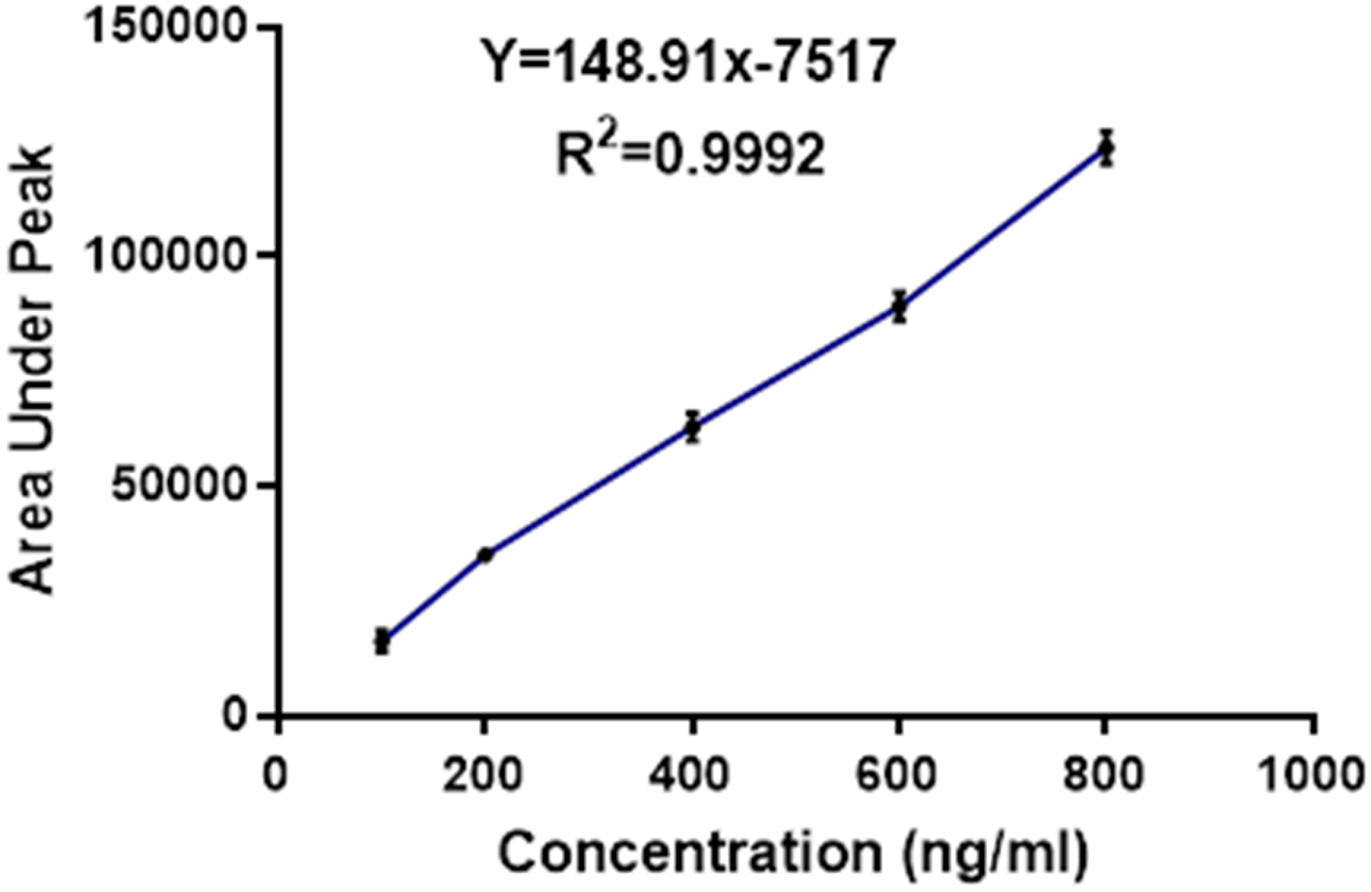
Calibration curve of the TC by HPLC.

**Figure 2:**
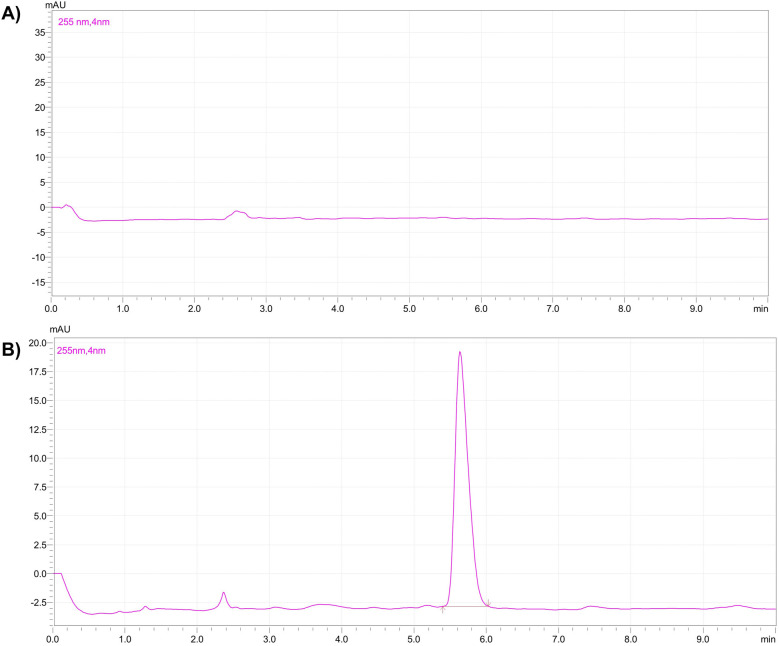
HPLC chromatogram of **(A)** blank plasma and **(B)** plasma spiked with TC.

After the dilution, each sample was mixed with an equal volume of organic solvent and vortexed for 10 min for liquid-liquid extraction. After 10 min, the organic layer was separated and evaporated to dryness. The samples were reconstituted in the 0.5 ml of mobile phase and, transferred into HPLC vials and subjected to the analysis (12). Figure 1 represents calibration curve of the TC by HPLC. Figure 2 represents HPLC chromatogram of (A) blank plasma and (B) plasma spiked with TC.

### 2.2 Chromatographic condition

The chromatographic condition for the analysis of the TC has been presented in Table 2.

**Table 2:** Demonstrating chromatographic condition for HPLC analysis.

HPLC model	SHIMADZU LC-20AR
Mobile phase	Acetonitrile and water (65:35)
Column	C18 SHIMADZU, 5 µm particle size, 25 cm length
Detector	Photodiode array detector
Lambda max (λmax)	255 nm
Run time	10 min
Retention time	5.7 min

### 2.3 Validation of the method

The developed method was validated by evaluating linearity, precision, accuracy, limit of detection (LOD), and limit of quantification (LOQ), which were done in acceptance of ICH and EMA guidelines.

#### 2.3.1 Linearity

The calibration plots were constructed by plotting peak areas against respective concentrations. Linearity was obtained by analysis of the serially diluted sample in the range of 100, 200, 400, 600, and 800 ng/ml.

#### 2.3.2 Precision

The degree of agreement within a set of measurements obtained from several samplings of the same homogenous sample under comparable analytical conditions is known as the precision of an analytical method (13). The acceptable values for% RSD should be within <5% at all concentration levels. The RSD was calculated as follows

%RSD=(Standarddeviation/meanobservedconcentration)×100

The precision of the method was further validated by assessing the recovery of Ticagrelor from samples and analyzing replicate injections of standard solutions.

#### 2.3.3 Accuracy

The accuracy of the method was determined by calculating% recovery (14). A known quantity of the drug was spiked in blank plasma at three different levels in triplicate over the specified concentration range and the amount of the drug was estimated by measuring the peak area ratio. The accuracy was also expressed as% bias, and the acceptable values should be within ± 15% at all concentration levels. The% bias was calculated as follows

$$\begin{array}{r} \text{\%}\;\mathrm{Bias}=(\mathrm{Observed}\;\mathrm{concentration}\text{-}\;\mathrm{Predicted}\;\mathrm{concentration}\;/\\ \mathrm{Predicted}\;\mathrm{Concentration})\times 100 \end{array}$$

The results of the accuracy study indicate that the analytical method consistently provided measurements that closely matched the reference values (15).

#### 2.3.4 LOD and LOQ

The LOD is defined as the lowest concentration of analyte that gives a detectable response. The limit of quantification LOQ is defined as the lowest concentration of analyte that can be quantified with a specified level of accuracy and precision. The LOD and LOQ were determined by injecting six replicates of analyte at progressively low concentrations of the standard solution using the developed HPLC methods (15).

The LOD of TC in the different mediums was calculated by using the following formula.

LOD=3.3×SD/S

Where SD is the standard deviation of y-intercept of the regression equation and S is the slope of the calibration curve. The LOQ of TC in different mediums was calculated by using the following formula

LOQ=10×SD/S

Where, SD is the standard deviation of the y-intercept of the regression equation and S is the slope of the calibration curve.

### 2.4 Ethical standards

The study was approved by the animal ethical committee NMIMS Shirpur campus, under reference no. SPTM/09/2023/IEAC/13. All protocols were followed according to the guidelines approved by the animal ethical committee. *In-vivo* studies were conducted with an appropriate number of animals, preferably in healthy Sprague Dawley rats.

### 2.5 Animals

The marketed or comparator formulation of the TC named as “Brilinta” used in this study was obtained from AstraZeneca. Healthy Sprague Dawley rats with an average weight of 250–350 g were used for the study. All the rats were subjected to standard food and environmental conditions. Rats were divided (Table 3) into 2 groups each containing 6 rats (*n* = 6).

**Table 3:** Represents animal grouping for the pharmacokinetic study.

Particulars	Details
Number of groups	2 groups (1 comparator and 1 formulation treatment)
Animal type	Healthy sprague dawley rats
Number of rats	6 (comparator) + 6 (formulation treatment) = 12 rats
Weight of animals	250–350 g
The dose of the drug to be administered	10 mg/kg
Blood sampling time points (hr)	0.5, 1, 2, 4, 8, 12 and 24 h
Parameters to be evaluated	T_max_ (hr) C_max_ (ng/ml) AUC_0–t_ (ng·h/ml) Half-life T_1/2_ (hr) MRT (hr) Vd (L) Relative bioavailability (%)

### 2.6 *In-vivo* pharmacokinetics study

The *in-vivo* pharmacokinetics study was performed in order to compare the plasma profile of the developed formulation and conventional formulation to establish the enhanced bioavailability of the developed formulation (16). All experiments were performed according to the guidelines approved by the animal ethical committee. *In vivo,* a pharmacokinetics study was conducted with an appropriate number of animals, preferably in healthy Sprague Dawley rats. The conventional TC and TC solid dispersion formulations corresponding to 10 mg/kg of TC were administrated to each animal via the oral route. The samples were dispersed in a carboxymethylcellulose solution prior to the administration. After the oral administration, blood sampling was done at 0.5, 1, 2, 4, 8, 12 and 24 h via retro-orbital plexus. Blood samples were centrifuged to collect the plasma. TC in the plasma samples was extracted with the help of ethyl acetate and was allowed for evaporation in distinct tubes; 300 μl of mobile phase was added to the dried tubes, then vortexed and subjected to centrifugation at 10,500 rpm for 15 min; 200 μl of the supernatant liquid was separated and transferred to HPLC vial inserts. The samples were analyzed by using developed HPLC analytical methods. The chromatographic condition includes the mobile phase of acetonitrile and water (65:35), 1 ml flow rate, 10 min run time, 5.8 retention time min, column C18 SHIMADZU (5 µm* 25 cm), photodiode array detector at 255 nm at λmax. The plasma samples of TC concentration were calculated by comparing them with the standard calibration curve of the TC (*R*^2^ = 0.9992).

### 2.7 Isolation of blood platelets and aggregation assays

Male Wistar rats were maintained in a temperature-regulated environment under a 12 h light-dark cycle. The rats were housed two per cage and fed for 10 weeks with either a conventional chow diet or a high-fat diet to induce obesity. This study was carried out to understand the platelet aggregation prevention capability of the developed formulation of the ticagrelor. A healthy group of rats fed with a conventional chow diet and disease group rat fed with high-fat diet. Additionally, 10 mg/kg dose of ticagrelor comparator and SD1, were simultaneously given along with high-fat diet, separately. Rats were anesthetized with isoflurane, and blood was collected in a 1:9 (v/v) ratio of ACD-C anticoagulant (12.4 mM sodium citrate, 13 mM citric acid, 11 mM glucose). Platelet-rich plasma (PRP) was obtained by centrifuging whole blood at 200 g for 15 min at ambient temperature. 5 ml of PRP was combined with 7 ml of washing buffer and centrifuged at 800 g for 13 min. The pellet was resuspended in a washing buffer, and this process was repeated once. The platelets were then gently suspended in Krebs solution, and the platelet count was adjusted to 1.2 × 10^8^ platelets/ml with the addition of 1 mM CaCl_2_. Platelet aggregation was measured using a dual-channel aggregometer at 37°C with stirring at 1,000 rpm. Aggregation was induced using ADP thrombin (100 mU/ml). Additionally, samples were observed under a brightfield microscope. The different groups were healthy control, disease control (high fat diet), Treatment group 1 (high fat diet + comparator), and Treatment group 2 (high fat diet + SD1).

### 2.8 Statistical analysis

All values in this study were presented as the Mean ± Standard Deviation (SD). The significant difference was evaluated with Prism 8 (GraphPad Software, CA, USA) according to student *t*-test) with the unpaired test. The level of the significance was denoted as; *P* > 0.05 (ns), *P* < 0.05 (Significant), *P* ≤ 0.01 (*), *P* ≤ 0.001 (**), *P* ≤ 0.0001 (***).

## 3 Result

### 3.1 Preparation of the calibration curve of the TC by HPLC method

The calibration curve for TC was successfully prepared using the HPLC method under the specified experimental condition. A series of TC standard solutions with concentrations ranging from 100 ng/ml to 800 ng/ml were injected into the HPLC system in duplicate, and corresponding peak areas were recorded. The calibration was constructed by plotting the concentration of TC (*x*-axis) against the peak area (*y*-axis), as shown in Table 4.

**Table 4:** Represents quantified area under the peak of the HPLC chromatogram.

TC concentration (ng/ml)	Area under peak
100	7,594
200	22,762
400	51,836
600	79,990
800	112,944

### 3.2 Validation of the methods

#### 3.2.1 Linearity

**Figure 3:**
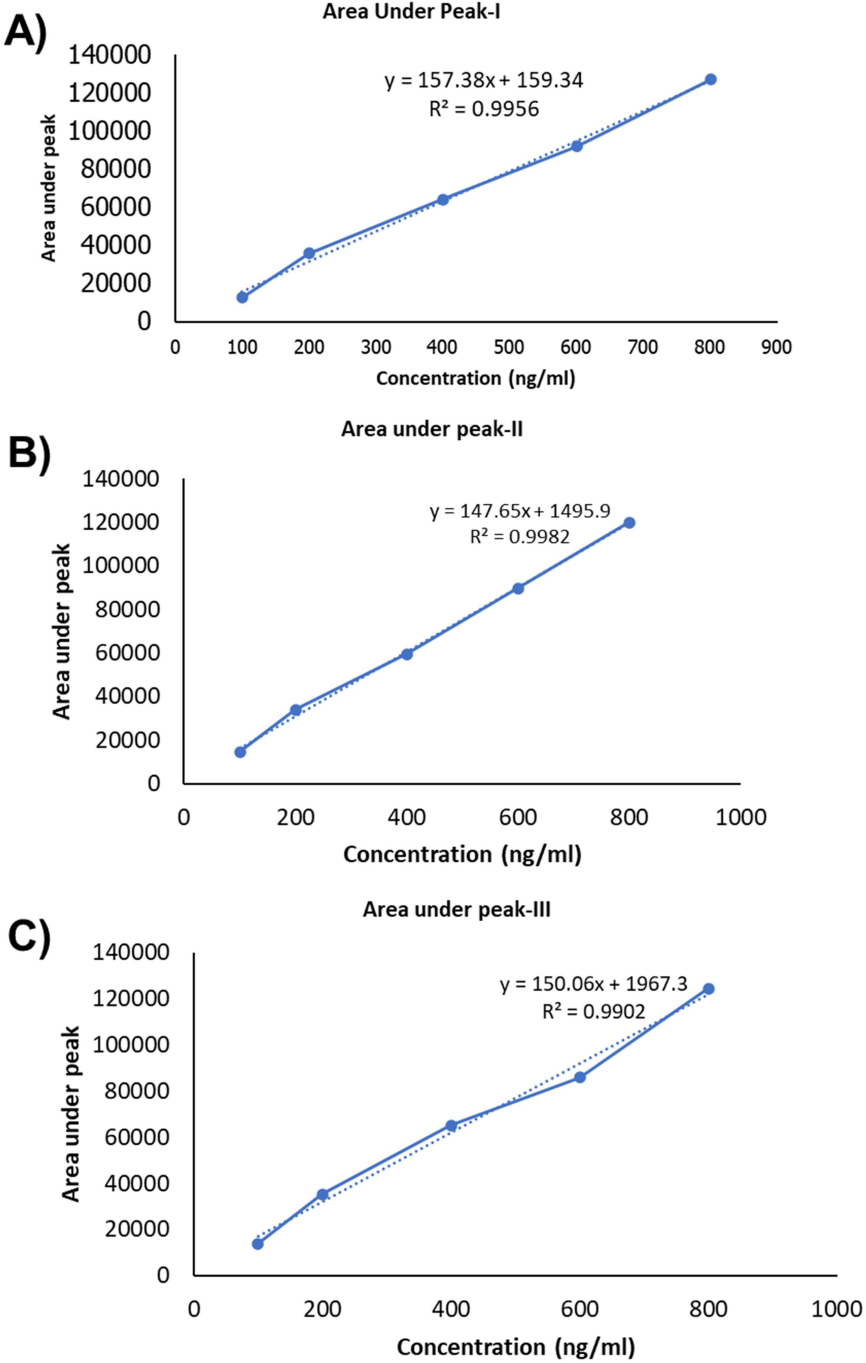
Linearity graph of the TC: **(A)** fisrt set of samples, **(B)** second set of samples and **(C)** third set of samples.

The resulting calibration curve exhibited Linearity (*R*^2^ = 0.995) over the concentration range studied, indicating a strong correlation between TC concentration and peak area. This suggests that the HPLC method used is suitable for the quantification of TC in unknown samples within the tested concentration range (17). Table 5 presents the drug concentration and respective areas under peak. Figure 3 represents the linearity graphs of TC in the range of 100-800 ng/ml. The calibration plots were constructed by plotting peak area against respective concentrations.

**Table 5:** Represents drug concentration versus area under peak.

Concentration(ng/ml)	Area under peak I	Area under peak II	Area under peak III
100	12,594	14,518	13,996
200	35,762	33,946	35,292
400	63,996	59,447	65,268
600	91,990	89,654	85,935
800	126,944	119,989	124,474
Slope	157.38	147.65	150.06
Intercept	159.34	1,495.90	1,967.30
STDDEV	45,166.01	42,320.62	43,184.13
STEYX	3,475.90	2,091.26	4,947.89

Linearity was obtained by analysis of the serially diluted sample in the range of 100, 200, 400, 600, and 800 ng/ml. Results are tabulated in the table as below.

#### 3.2.2 Precision

The accuracy and precision of the method were further validated by assessing the recovery of TC from spiked samples and analyzing replicate injections of standard solutions. The relative standard deviation (RSD) for replicate injection was less than 5%, demonstrating the method's precision (18). The% RSD values for the calculated for 100–800 ng has been presented in Table 6.

**Table 6:** Demonstrating the calculated RSD value for the precision.

–	100 ng	200 ng	400 ng	600 ng	800 ng
STD	994.98	942.56	3,060.38	3,053.71	3,525.81
%RSD	4.26	2.69	4.87	3.42	2.85

#### 3.2.3 Accuracy

The results of the accuracy study indicate that the analytical method consistently provided measurements that closely matched the reference values. The percentage recovery, which quantifies the method's accuracy, was calculated for each value (19). All the individual measurements show percentage recoveries within an acceptable range (Table 7).

**Table 7:** Demonstrating the calculated recovery for determining accuracy.

Concentration (ng/ml)	Amount recovered (peak area-1)	Amount recovered (peak area-1)	Amount recovered (peak area-1)
100	79.01	88.20	80.16
200	226.22	219.78	222.08
400	405.62	392.49	421.84
600	583.50	597.07	559.56
800	805.60	802.53	816.38

#### 3.2.4 LOD and LOQ

The assessment of LOD and LOQ underscores the sensitivity and performance capabilities of the analytical method. These values are crucial for ensuring for ensuring that the method is fit for its intended purpose, particularly in applications where TC concentrations may be near or below these limits. LOD for peak I–III were 72.88, 46.74, 108.8, respectively and LOQ for peak I–III were 220.86, 141.64, and 329.73, respectively. The variation in the LOD and LOQ values among replicates may be due to the instrumental sensitivity, drug properties, matrix effect, chromatographical conditions and experimental errors (20).

### 3.3 *In-vivo* pharmacokinetics study

These studies provide critical information about the drug's absorption, distribution, metabolism, and excretion (ADME) properties (21). Specific methods of statistical experimental sections were applied to evaluate the patterns and trends in data.

All values in this study were presented as the Mean ± Standard Deviation (SD). The significant difference was evaluated with Prism 8 (GraphPad Software, CA, USA) according to student *t*-test) with the unpaired test. The level of the significance was denoted as; *P* > 0.05 (ns)_, *P* < 0.05 (Significant), *P* ≤ 0.01(*), *P* ≤ 0.001(**), *P* ≤ 0.0001(***). These studies provide critical information about the drug's absorption, distribution, metabolism, and excretion. The specific results of *in-vivo* studies pharmacokinetic studies can vary on different parameters such as Tmax, Cmax, Half-life, AUC, Volume of distribution, and Relative bioavailability. The results of *in-vivo* studies are shown in Table 8.

**Table 8:** Pharmacokinetic profile of the TC, comparator, SD1 .

Pharmacokinetic parameters	Comparator (RLD)	SD1
C_max_ (ng/ml)	3,954.04 ± 93.92	5,381.675 ± 416.60
AUC_tot_ (ng/ml*h)	46,975.80 ± 927.45	77,196.698 ± 427.44
T_1/2_ (hr)	10.26 ± 1.51	9.216 ± 0.97
MRT (hr)	15.66 ± 1.624	14.059 ± 1.56
Vd (L)	0.49 ± 0.07	0.29 ± 0.02
Relative bioavailability (%)	–	64.33 ± 3.40

**Figure 4:**
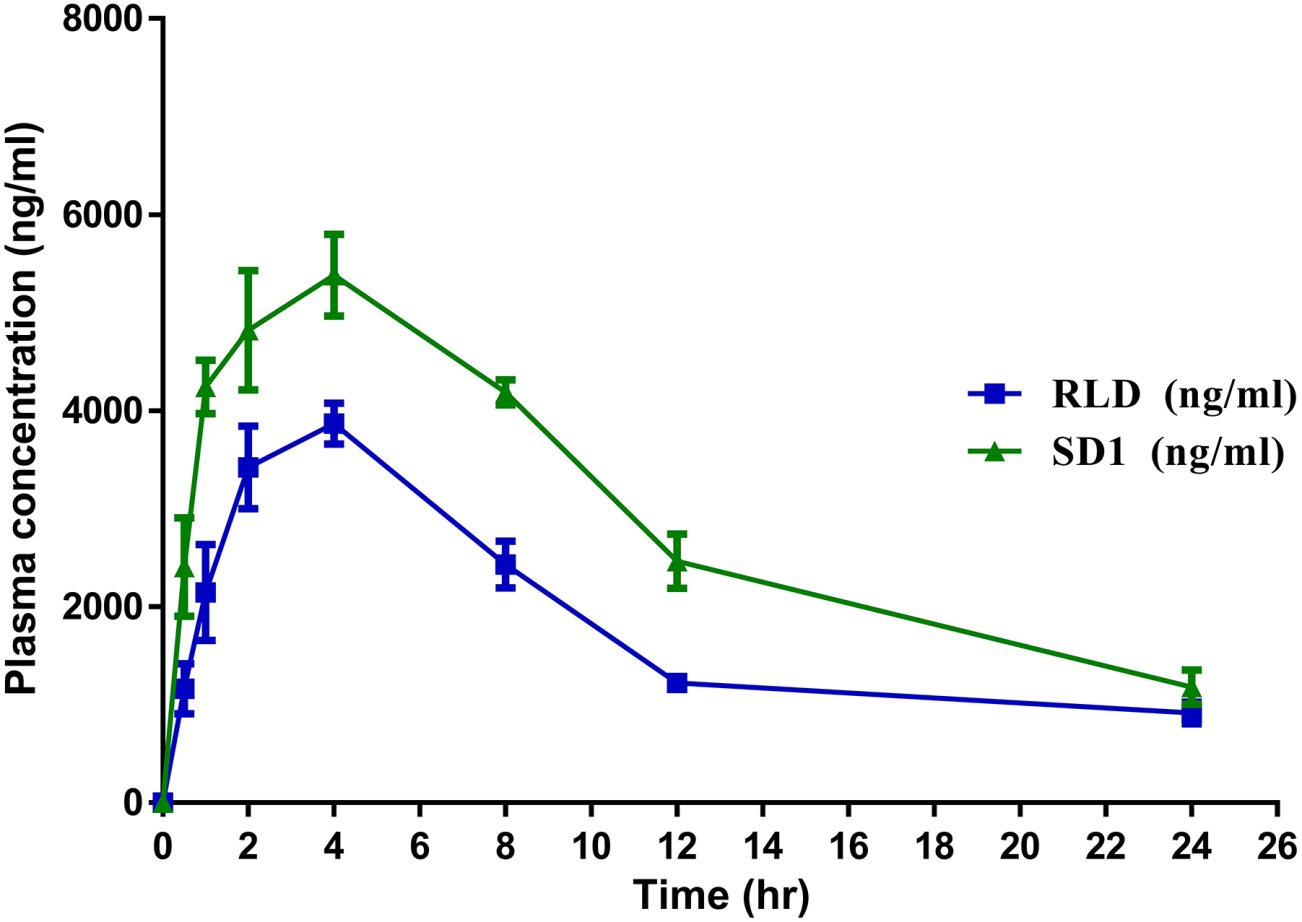
Plasma concentration time profile of the comparator and SD1.

The *in-vivo* pharmacokinetic study demonstrated that after the development of the solid dispersion formulation^11^, there was a significant enhancement in C_max_ (*P* ≤ 0.001), AUC (*P* ≤ 0.0001), and relative bioavailability (*P* ≤ 0.001) compared to the conventional marketed Ticagrelor (TC) Tablet. Notably, the bioavailability of the developed TC amorphous solid dispersion formulation was improved by up to 64.33% (*P* < 0.0001). There was a significant reduction in Vd (*P* ≤ 0.001), which indicates reduced nonspecific delivery of the drug to lipid compartment of the body space and indirectly indicates enhancement in bioavailability. Whereas, there was no significant difference between T_1/2_ and MRT (*P* ≥ 0.05). Figure 4 represents plasma concentration time profile of the comparator and SD1.

The pharmacokinetic profile Ticagrelor [Comparator Vs Amorphous Solid Dispersion] has been demonstrated in the Table 8.

### 3.4 Isolation of blood platelets and aggregation assays

Ticagrelor reduces platelet aggregation by antagonizing the P_2_Y_12_ receptor, which plays a key role in ADP-induced platelet activation and aggregation. In this, investigation, inhibition of the platelet's aggregation induced by ADP thrombin were evaluated for RLD and SD1 formulation. Platelet aggregation is one of the key steps in the formation of the clot; ticagrelor is supplied as a drug that inhibits the aggregation of the platelets and hence prevents clot formation.

**Figure 5:**
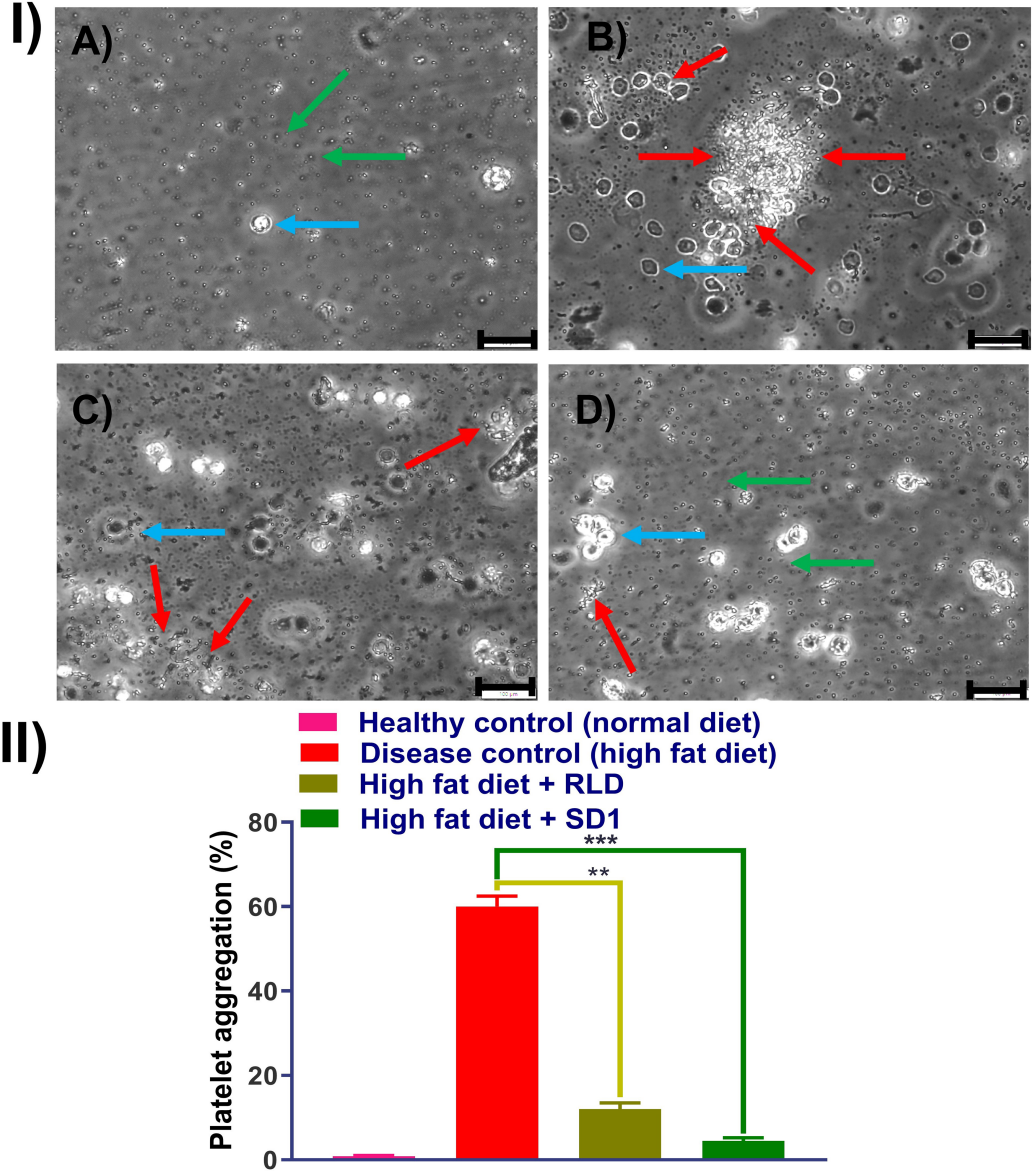
Effect of platelet aggregation inhibition effect of RLD and SD1, (I) bright filed image of plate obtained from **(A)** healthy rat, **(B)** high fat diet rat, **(C)** high fat diet rat + RLD, **(D)** high fat diet rat + SD1, and (II) % platelet aggregation measured dual-channel aggregometer. (Green arrow: individual platelets, red arrow: aggregated platelets, Capri blue arrow: RBCs).

In Figure 5IA, demonstration the platelets free from, as healthy control, whereas Figure 5IB, platelets were aggregated following treatment with ADP thrombin (disease control). The RLD-treated group showed fewer platelet aggregation and, which was further reduced in SD1 treated group. Additionally, data obtained from the platelet aggregometer (Figure 5II), depicted that SD1 treated group had a lower% of platelet aggregation compared to the RLD. Hence, our developed formulation (SD1) demonstrated better platelet aggregation protection compared to the marketed formulation of ticagrelor. Overall, it was observed that when ticagrelor formulation was given to the rats (orally), it reduced the plate aggregation by binding to the P_2_Y_12_ receptor.

## 4 Discussion

The present study aimed to enhance the oral bioavailability of TC through the development of a solid dispersion formulation, subsequently evaluated using both *in-vitro* and *in-vivo* approaches. The calibration curve preparation via HPLC ensured accurate quantification of TC within the specified concentration range, with linearity demonstrated across concentrations ranging from 100 ng/ml to 800 ng/ml. The robustness of the method was evidenced by the strong correlation (*R*^2^ = 0.995) between TC concentration and peak area, suggesting suitability for routine analysis. In a study, Kim et al, reported that solid dispersion of the TC prepared by solvent evaporation method enhanced oral bioavailability and intestinal permeability of the TC, however, they have not compared with the marketed formulation (8).

Validation studies further confirmed the reliability of the HPLC method. Precision analysis indicated low % RSD values for replicate injections, underscoring the method's repeatability and reliability. Similarly, accuracy assessment revealed percentage recovery within an acceptable range, validating the method's ability to provide measurements closely matching the reference values. Furthermore, LOD and LOQ determination emphasized the method's sensitivity, which is crucial for detecting low concentrations of TC with precision and accuracy.

*In-vivo* pharmacokinetic studies provided critical insights into the comparative performance of the developed TC formulation against the innovator-marketed tablet. The solid dispersion formulation exhibited enhanced pharmacokinetic parameters, notably increased Cmax and AUC, indicative of improved systemic exposure. The observed reduction in T_1/2_ and MRT suggests accelerated drug clearance, possibly attributed to the enhanced solubility and bioavailability of TC in the solid dispersion formulation. In a study, Yadav et al, developed solvent free solid dispersion for improving solubility and dissolution of the TC. Developed formulation was found to be higher permeable and had better bioavailability compared to the pure TC (22).

Notably, the relative bioavailability of the developed TC formulation was significantly improved by approximately 64% compared to the innovator product. This enhancement underscores the potential clinical benefits of the solid dispersion formulation, potentially leading to improved therapeutic outcomes in patients requiring Ticagrelor therapy. The plasma concentration-time profiles further illustrate the distinct pharmacokinetic profiles of the comparator and the solid dispersion formulation, reaffirming the formulation's efficacy in enhancing drug absorption and systemic exposure.

Ticagrelor reduces platelet aggregation by binding to the P_2_Y_12_ receptor. In this study, the SD1 formulation showed greater inhibition of ADP-induced platelet aggregation compared to the marketed ticagrelor (RLD) formulation. This was mainly due to enhancement in the solubility and bioavailability of the developed formulation compared to the RLD (23).

## 5 Conclusion

The *in-vivo* pharmacokinetics studies play a pivotal role in the field of pharmacology and drug development. These studies involve meticulous experimentation, often in animals or human subjects, and require adherence to ethical and regulatory guidelines. These studies are fundamental components of the drug development process, offering essential data to make informed decisions about a drug's safety, efficacy, and optimal usage. The developed HPLC method was found to be simple, precise, accurate and sensitive for the estimation of TC. Validation results was, according to ICH and EMA carried out revealed high accuracy and good precision. Following dosing rats with formulation and blood samples were collected, and plasma was separated. The drug concentration in the plasma was evaluated by validated HPLC methods. The pharmacokinetic parameters were calculated, it was observed that our developed formulation has improved pharmacokinetic profile with enhancement in bioavailability. The improvement in bioavailability was observed up to 64% relative to the marketed formulation.

This study is limited to the animal model and single dosing, however, in the future, it can be extended to clinical trials.

## Data Availability

The original contributions presented in the study are included in the article/Supplementary Material, further inquiries can be directed to the corresponding author.
